# Association between estimated glucose disposal rate and the risk of atherosclerotic cardiovascular disease: insight from cross-sectional and retrospective cohort studies

**DOI:** 10.3389/fnut.2025.1664591

**Published:** 2025-12-10

**Authors:** Zixuan Wang, Qingyun Zhou, Tao Zheng, Taotao Huang, Kefan Yi

**Affiliations:** 1 Department of Vascular Surgery, Ningbo Medical Center Lihuili Hospital, Ningbo University, Ningbo, China; 2 Department of Clinical Nutrition, Shanghai Deji Hospital, Qingdao University, Shanghai, China

**Keywords:** atherosclerotic cardiovascular disease, estimated glucose disposal rate, cross-sectional study, retrospective cohort study, mediation analysis

## Abstract

**Background:**

Accumulating data have elucidated a significant association between insulin resistance (IR) and the risk of cardiovascular diseases. The estimated glucose disposal rate (eGDR), a quantitative indicator of glucose metabolism, has been increasingly recognized as a reliable clinical indicator of IR. Atherosclerotic cardiovascular disease (ASCVD) is a type of cardiovascular disease caused by atherosclerosis. However, the association and potential mechanisms between eGDR and ASCVD risk remain unclear.

**Methods:**

We conducted cross-sectional and retrospective cohort studies using data from the two health examination centers of Qingdao University Affiliated Shanghai Deji Hospital and Ningbo Medical Center Lihuili Hospital to horizontally and longitudinally evaluate the potential relationship and explore potential mechanisms between eGDR and the risk of ASCVD.

**Results:**

A significant negative linear association between eGDR and ASCVD risk was observed in our study. After adjusting for some covariates, each unit increment in eGDR was associated with a 16.1% decrease in odds ratio of ASCVD (OR = 0.839, 95% CI: 0.806–0.894) in the cross-sectional study and a 12.4% decrease in hazard ratio of ASCVD (HR = 0.876, 95% CI: 0.788, 0.974) in the retrospective cohort study. This negative association was robust in most subgroups and various sensitivity analyses. Furthermore, eGDR presented greater predictive performances for ASCVD diagnosis and new-onset ASCVD risk compared with TyG, HOMA-IR, and METS-IR. In addition, AIP, VAI, CMI, LAP, and BMI had partial mediation effects on the association between eGDR and ASCVD risk.

**Conclusion:**

This study elucidates the significant negative dose-response linear relationship of eGDR with ASCVD risk and the partial mediation effects of AIP, VAI, CMI, LAP, and BMI, highlighting the significance of enhancing glucose-utilizing capacity in decreasing the risk of ASCVD.

## 1 Introduction

Atherosclerotic cardiovascular disease (ASCVD), which constitutes a spectrum of vascular pathologies including coronary, cerebral, and peripheral arterial manifestations, has epidemiologically emerged as the leading global burden of cardiovascular disease and fatal outcomes worldwide (1, 2). In the USA, ASCVD accounts for 28–43% of mortality in cardiovascular diseases (3, 4). Some studies have demonstrated that hypertension, hyperlipidemia, smoking, and diabetes are established risk factors in improving the occurrence and progression of ASCVD risk. However, there are no exact etiological mechanisms for the occurrence and progression of ASCVD. In addition, recent evidence has demonstrated that insulin resistance (IR) may be an important risk factor for increasing the risk of ASCVD (5). The study of Shi et al. demonstrated that HOMA-IR and METS-IR, well-established biomarkers of IR, exhibit a significant dose-response relationship with ASCVD risk (6). However, the potential mechanisms of the association between IR and ASCVD remain incompletely elucidated, and it is imperative to further explore possible mechanisms using large population research.

Insulin resistance (IR) represents a fundamental endocrine dysfunction wherein target tissues demonstrate impaired sensitivity to physiological insulin action at both receptor and post-receptor levels, constituting a core metabolic disorder in numerous chronic diseases (7, 8). This metabolic disorder is primarily manifested through impaired glucose homeostasis, particularly in skeletal muscle and adipose tissue, resulting from defective insulin-stimulated glucose uptake and utilization (9, 10). The progression of IR has been shown to involve multifactorial etiology, with genetic predisposition and environmental determinants synergistically contributing to its pathogenesis through complex gene-environment interactions (11, 12). The estimated glucose disposal rate (eGDR), calculated by waist circumference and hypertension status, hemoglobin A1c (HbA1c), has emerged as a validated surrogate marker for IR (13, 14). When eGDR levels increased, glucose utilization ability is enhanced, which suggests a low risk of IR. Therefore, eGDR demonstrates significant clinical utility and predictive value in the risk of IR (15, 16). Currently, some evidence has elucidated a significant negative dose-dependent association between high eGDR levels and incident adverse cardiovascular disease, particularly myocardial infarction and ischemic stroke. However, the relationship of eGDR with ASCVD risk remains unclear. In addition, some epidemiological studies have demonstrated that obesity status is linked with both eGDR and ASCVD (17–19). For example, Geng et al. demonstrate that eGDR was negatively associated with WWI, as an obesity related index, in two cohort studies in China and the UK (20). In addition, Makhmudova et al. used UK Biobank data to explore the association between visceral obesity and risk of ASCVD, and their findings reveal that high visceral obesity increased the risk of ASCVD (21). Therefore, based on the above evidence, we speculate that obesity may be a possible mediating factor in the association of eGDR with ASCVD risk. However, no studies have yet investigated the potential mediating effect of obesity in the association of eGDR with ASCVD risk.

To elucidate this relationship and potential mechanisms, we conducted cross-sectional and retrospective cohort studies using population health examination data from the health examination centers of Qingdao University Affiliated Shanghai Deji Hospital and Ningbo Medical Center Lihuili Hospital to horizontally and longitudinally examine the association of eGDR with ASCVD risk and investigate the potential mediating effects of five obesity-related biomarkers, including the atherogenic index of plasma (AIP), visceral adiposity index (VAI), cardiometabolic index (CMI), lipid accumulation product (LAP), and body mass index (BMI).

## 2 Materials and methods

### 2.1 Study design and participants

We designed a cross-sectional study using population health examination data from June 1, 2011, to June 1, 2012, in the two health examination centers of Qingdao University Affiliated Shanghai Deji Hospital and Ningbo Medical Center Lihuili Hospital to horizontally explore the association of eGDR and the risk of ASCVD. Furthermore, we also conducted a retrospective cohort study by following up four times with individuals without ASCVD from June 6, 2012, to December 31, 2024 to longitudinally explore the association of eGDR and new-onset ASCVD risk. The average follow-up time for these individuals was 8.2 years. Individuals with newly diagnosed ASCVD were the primary outcome in our retrospective cohort study. This cross-sectional and retrospective cohort studies were approved by the Ethics Committee of the Qingdao University Affiliated Shanghai Deji Hospital (SHDJ-2025-10) and Ningbo Medical Center Lihuili Hospital (NBLHLH-2025-15). Each individual voluntarily signed informed consent documents in accordance with the Declaration of Helsinki principles.

**FIGURE 1:**
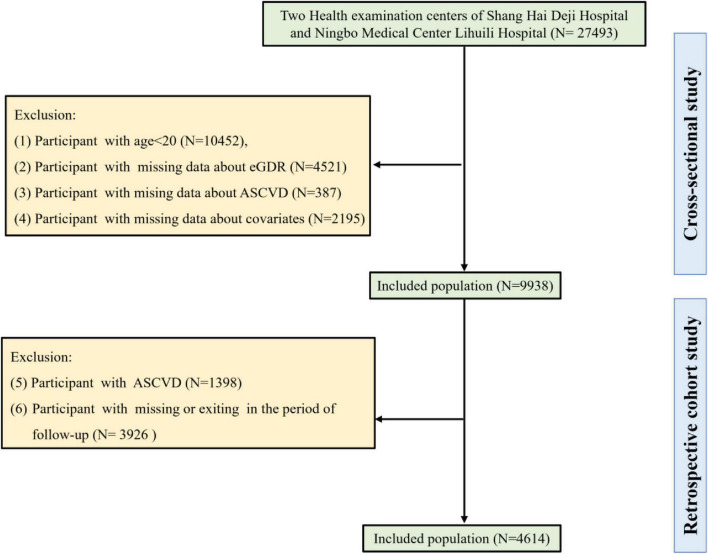
Participant flow chart showing inclusion and exclusion criteria.

Our cross-sectional study initially included 27,493 individuals from two health examination centers of Qingdao University Affiliated Shanghai Deji Hospital and Ningbo Medical Center Lihuili Hospital. According to the exclusion criteria of our cross-sectional study, individuals under 20 years of age (*N* = 10,452), those with missing eGDR data (*N* = 4,521), missing ASCVD data (*N* = 387), and missing covariate information (*N* = 2,195) were excluded. Finally, 9,938 individuals were included in our cross-sectional study. In addition, 4,614 participants were included in our retrospective cohort study after excluding individuals with ASCVD (*N* = 1,398), and those with missing or exiting in the period of follow-up (*N* = 3,926). A detailed flowchart (Figure 1) delineated the participant selection process.

### 2.2 Measurement of the eGDR

The eGDR was calculated using a multivariable-adjusted algorithm: 21.158− (0.09 × WC) − (3.407 × HT) − (0.551 × HbA1c); incorporating: WC (waist circumference in cm), HT (hypertension status defined as 1 = present, 0 = absent), and HbA1c (glycated hemoglobin measured in %) (22).

### 2.3 Definition of ASCVD

Following the American Heart Association guidelines and established clinical criteria, ASCVD was defined as: participants with physician-diagnosed cardiovascular conditions, including coronary artery disease, angina pectoris, myocardial infarction, cerebrovascular accident, congestive heart failure, and stroke (23).

### 2.4 Measurement of AIP, VAI, CMI, LAP, and BMI

The related obesity indices were calculated as the following formulas, respectively:

AIP = log10 [TG (mg/dL)/HDL-C (mg/dL)].
VAI (male) = [WC (cm)/39.68 + (1.88 × BMI)] × [TG (mmol/L)/1.03] × [1.31/HDL − C (mmol/L)].
VAI (female) = [WC (cm)/36.58 + (1.89 × BMI)] × [TG (mmol/L)/0.81] × [1.52/HDL − C (mmol/L)].
CMI = [TG (mmol/L)/HDL-C (mmol/L)] × [WC (cm)/height (cm)],
LAP (male) = [WC (cm) − 65] × [TG (mg/dL)], LAP (female) = [WC (cm) − 58] × [TG (mg/dL)], BMI = [weight (kg)/height^2^ (m)] (24–28).

### 2.5 Covariates

Our study included the following sociodemographic, lifestyle, and clinical biomarker covariates. Sociodemographic and lifestyle covariates included age (<60 years, ≥ 60 years), gender (female and male), education levels (less than high school, high school, more than college), and poverty index ratio (PIR) (< 1.0, 1.0–3.0, and ≥ 3.0), body mass index (BMI) (< 18.5, 18.5–24.9, 25–29.9, ≥ 30 kg/m^2^), smoking status (never/former/current smoker) and drinking status (never/former/current drinker), physical activity (vigorous/moderate/low level). Current smoker was regarded as participants with a lifetime cumulative smoking of ≥ 100 cigarettes, and were still smoking at the time of the survey. Former smoker was regarded as participants with a lifetime cumulative smoking of ≥ 100 cigarettes, but had quit smoking at the time of the survey. Never smoker was regarded as participants with a lifetime cumulative smoking of ≤ 100 cigarettes (29). Current drinker was determined as participants who have consumed any alcoholic beverage within the past 12 months. Former drinker was determined as participants who previously drank regularly but have abstained from alcohol for more than 12 months. Never drinker was determined as participants with a lifetime alcohol consumption < 12 drinks (30). Vigorous activity refers to engaging in vigorous physical activity at least 3 days per week, accumulating a total of at least 1,500 MET-minutes per week. Moderate activity refers to engaging in at least 30 min of moderate-intensity activity or brisk walking on at least 5 days per week. Low-level activity refers to those who do not meet the standards for “moderate” or “Vigorous” levels (31, 32). Hypertension was defined as participants with three times average systolic blood pressure (SBP) of ≥ 130 and a diastolic blood pressure (DBP) of ≥ 80 mmHg, or diagnosed by a physician, or usage of lower blood pressure medicine, which was categorized into yes or no. Diabetes was defined as participants with fasting glucose ≥ 126 mg/dL or HbA1c ≥ 6.5%, or diagnosis by a physician, or usage of lower blood glucose medicine, which was categorized into yes or no (33, 34). Clinical biomarkers covariates included waist circumference (WC), total cholesterol (TC), triglyceride (TG), low-density lipoprotein cholesterol (LDL), high-density lipoprotein cholesterol (HDL), glycated hemoglobin A1c (HbA1c), fast glucose, C-reactive protein (CRP), uric acid (UA), total cholesterol (TC), albumin (ALB), aspartate aminotransferase (AST), alanine aminotransferase (ALT), gamma-glutamyl transferase (GGT), and lactate dehydrogenase (LDH).

### 2.6 Statistical analysis

**FIGURE 2:**
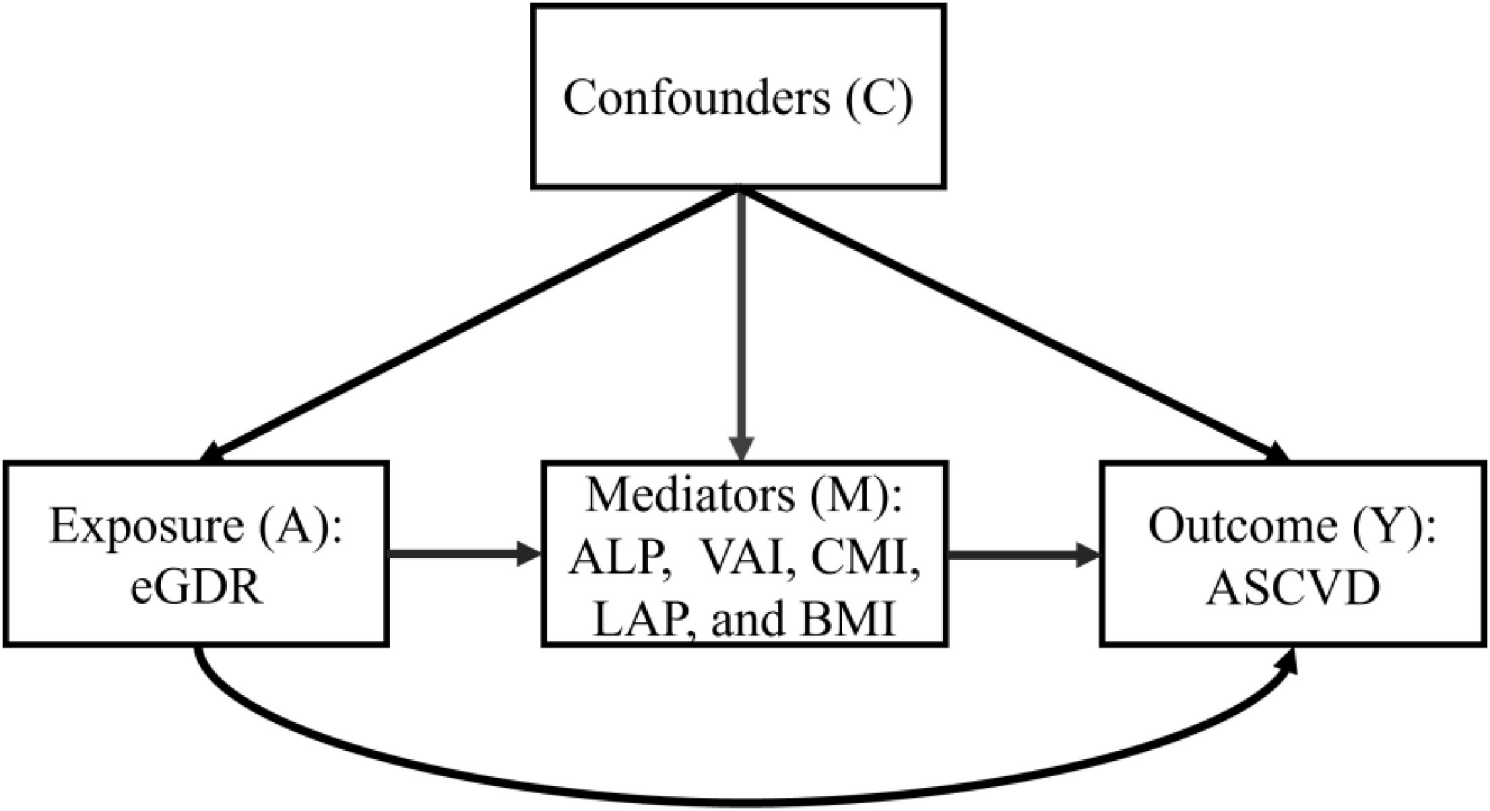
Causal framework of exposure and outcome.

In this study, statistical analysis was performed on the baseline characteristics of the participants, using Student’s *t*-test for continuous variables and χ^2^ tests for categorical variables. The eGDR was evaluated as both continuous and categorical variables, with quartile-based stratification (Q1–Q4) applied for categorical analyses. To assess potential associations between eGDR and ASCVD risk, we conducted multivariable logistic regression models, a multivariable Cox proportional hazards regression model, and restricted cubic spline (RCS) regression models. The predictive performance of eGDR for ASCVD diagnosis was evaluated using receiver operating characteristic (ROC) curve analysis, C-statistic index, net reclassification improvement (NRI), and integrated discrimination improvement (IDI). The predictive value of eGDR for new-onset ASCVD risk was evaluated using a time-dependent receiver operating characteristic (Time-dependent ROC) curve, C-index, NRI, and IDI. To evaluate the robustness of the association of eGDR with ASCVD risk, we conducted subgroup analyses and sensitivity analyses, such as using multiple imputations for missing covariate data, deleting participants with hypertension, diabetes, and chronic kidney disease, depression, and cancer, and using Fine-Gray competing-risk models that included deaths of non-ASCVD as competing events. We used the mediation analysis repeated bootstrap sampling method (*N* = 5,000) to explore the mediation effect of obesity-related indices (AIP, VAI, CMI, LAP, and BMI). The causal framework was shown in Figure 2. To control the potential type I error, multiple testing corrections were performed through the Bonferroni correction method. All statistical analyses were performed using R software (version 4.3.3). *P* < 0.05 was used as the statistical significance criterion.

## 3 Results

### 3.1 Baseline demographic characteristics of the participants

Table 1 and Supplementary Table 1 summarized the baseline characteristics of the participants. As shown in Table 1, this cross-sectional study included 9,938 participants aged ≥ 20 years, among whom 52.11% (*n* = 5,179) were female, and 14.07% (*n* = 1,398) met diagnostic criteria for ASCVD. In addition, our retrospective cohort study comprised 4,614 participants, among whom 47.88% (*n* = 2,209) were female, and 18.80% (*n* = 868) were new-onset ASCVD (Supplementary Table 1). Significant differences (*P* < 0.05) were observed across all covariates when comparing participants with ASCVD to those with non-ASCVD in cross-sectional and retrospective cohort studies. In addition, the baseline demographic characteristics of the included and excluded participants were displayed in Supplementary Table 2, and no significant differences (*P* > 0.05) were observed across most covariates when comparing included participants to those excluded in the retrospective cohort study.

**TABLE 1:** Baseline demographic characteristics of the participants in the cross-sectional study.

Variables	Total (*n* = 9,938), *n* (%) or mean (SE)	ASCVD (*n* = 1,398), *n* (%) or mean (SE)	None- ASCVD (*n* = 8,540), *n* (%) or mean (SE)	SMD or ASD	*P*-value
Age	51.91 (17.11)	57.02 (16.01)	50.33(17.22)	0.780	<0.001
Gender					<0.001
Female	5,179 (52.11)	695 (49.79)	4,484 (52.50)	0.027	
Male	4,759 (47.89)	703 (50.21)	4,056 (47.50)	0.027	
Educational levels					<0.001
Less than high school	1,363 (13.72)	449 (32.12)	914 (10.70)	0.214	
High school	2,204 (22.18)	595 (42.56)	1,609 (18.84)	0.237	
College or above	6,371 (64.10)	354 (25.32)	6,017 (70.45)	0.451	
PIR					<0.001
PIR < 1	1,598 (16.08)	419 (29.97)	1,179 (13.81)	0.161	
1 ≤ PIR < 3	3,910 (39.34)	566 (40.49)	3,344 (39.16)	0.013	
PIR > 3	4,430 (44.57)	413 (29.54)	4,017 (47.03)	0.174	
BMI					<0.001
Underweight	110 (1.11)	25 (1.79)	85 (1.00)	0.008	
Normal weight	2,400 (24.15)	412 (29.47)	1,988 (23.28)	0.062	
Overweight	3,200 (32.20)	382 (27.32)	2,818 (33.00)	0.057	
Obesity	4,228 (42.54)	579 (41.42)	3,649 (42.72)	0.013	
Drinking status					<0.001
Never drinker	811 (8.16)	196 (14.02)	615 (7.20)	0.068	
Former drinker	2,750 (27.67)	583 (41.70)	2,167 (25.37)	0.163	
Current drinker	6,377 (64.17)	619 (44.28)	5,758 (67.43)	0.231	
Smoking status					<0.001
Never smoker	4,764 (58.00)	513 (36.70)	4,251 (49.78)	0.131	
Former smoker	2,526 (25.41)	390 (27.90)	2,136 (25.01)	0.029	
Current smoker	2,648 (16.59)	495 (35.40)	2,153 (25.21)	0.102	
Physical activity					<0.001
Vigorous level	3,450 (34.72)	242 (17.31)	3,208 (37.57)	0.203	
Middle level	2,861 (28.78)	708 (50.64)	2,153 (25.21)	0.254	
Low level	3,627 (36.50)	448 (32.05)	3,179 (37.22)	0.051	
Hypertension					<0.001
Yes	4,700 (47.30)	752 (53.79)	3,948 (46.23)	0.076	
No	5,238 (52.70)	646 (46.21)	4,592 (53.77)	0.076	
Diabetes					<0.001
Yes	1,928 (19.40)	768 (54.94)	1,160 (13.58)	0.414	
No	8,010 (80.60)	630 (45.06)	7,380 (86.42)	0.414	
eGDR (mg/kg/min)	6.89 (2.61)	5.60 (2.51)	7.29 (2.78)	0.863	<0.001
WC (cm)	98.42 (7.46)	105.17 (8.15)	92.12 (6.79)	0.735	<0.001
TC (mg/dL)	231.45 (5.24)	236.23 (5.89)	228.48 (5.13)	0.920	<0.001
TG (mg/dL)	135.03 (6.12)	138.23 (6.58)	132.79 (5.69)	0.303	<0.001
HDL (mg/dL)	39.14 (4.32)	37.28 (4.78)	41.01 (4.13)	0.807	<0.001
HbA1c (%)	5.13 (5.03)	5.89 (5.34)	4.71 (4.68)	0.562	<0.001
Fast glucose (mg/dL)	94.02 (7.13)	99.35 (8.02)	91.32 (6.25)	0.345	<0.001
CRP (mg/L)	2.84 (1.56)	3.25 (1.49)	2.67 (1.72)	0.691	<0.001
UA (mg/dL)	5.96 (2.38)	6.34 (2.51)	5.72 (2.22)	0.814	<0.001
AST (U/L)	23.14 (4.34)	24.62 (4.89)	22.06 (4.05)	0.426	<0.001
ALB (g/L)	45.23 (4.31)	48.16 (4.98)	43.03 (4.06)	0.753	<0.001
ALT (U/L)	24.00 (4.00)	27.0 (5.00)	22.0 (3.00)	0.464	<0.001
GGT (U/L)	36.16 (3.34)	38.19 (3.59)	34.65 (3.12)	0.791	<0.001
LDH (U/L)	143.27 (7.03)	145.48 (7.34)	139.98 (6.89)	0.555	<0.001

SMD, standardized mean difference; ASD, absolute standardized difference; PIR, family income-to-poverty ratio; BMI, body mass index; WC, waist circumference; TC, total cholesterol; TG, triglyceride; LDL, low-density lipoprotein cholesterol; HDL, high-density lipoprotein cholesterol; HbA1c, glycated hemoglobin A1c; CRP, C-reactive protein; UA, uric acid; ALB, albumin; ALT, alanine aminotransferase; AST, aspartate aminotransferase; GGT, gamma-glutamyl transferase; LDH, lactate dehydrogenase; ASCVD, atherosclerotic cardiovascular disease. Categorical variables were presented as % and ASD, with χ^2^; tests comparing statistical differences between groups. Continuous variables were reported as mean ± standard error (SE) and SMD, with Student’s *t*-test in between-group comparisons. *P* < 0.05 was regarded as statistically significant.

### 3.2 Association between eGDR and the risk of ASCVD

**FIGURE 3:**
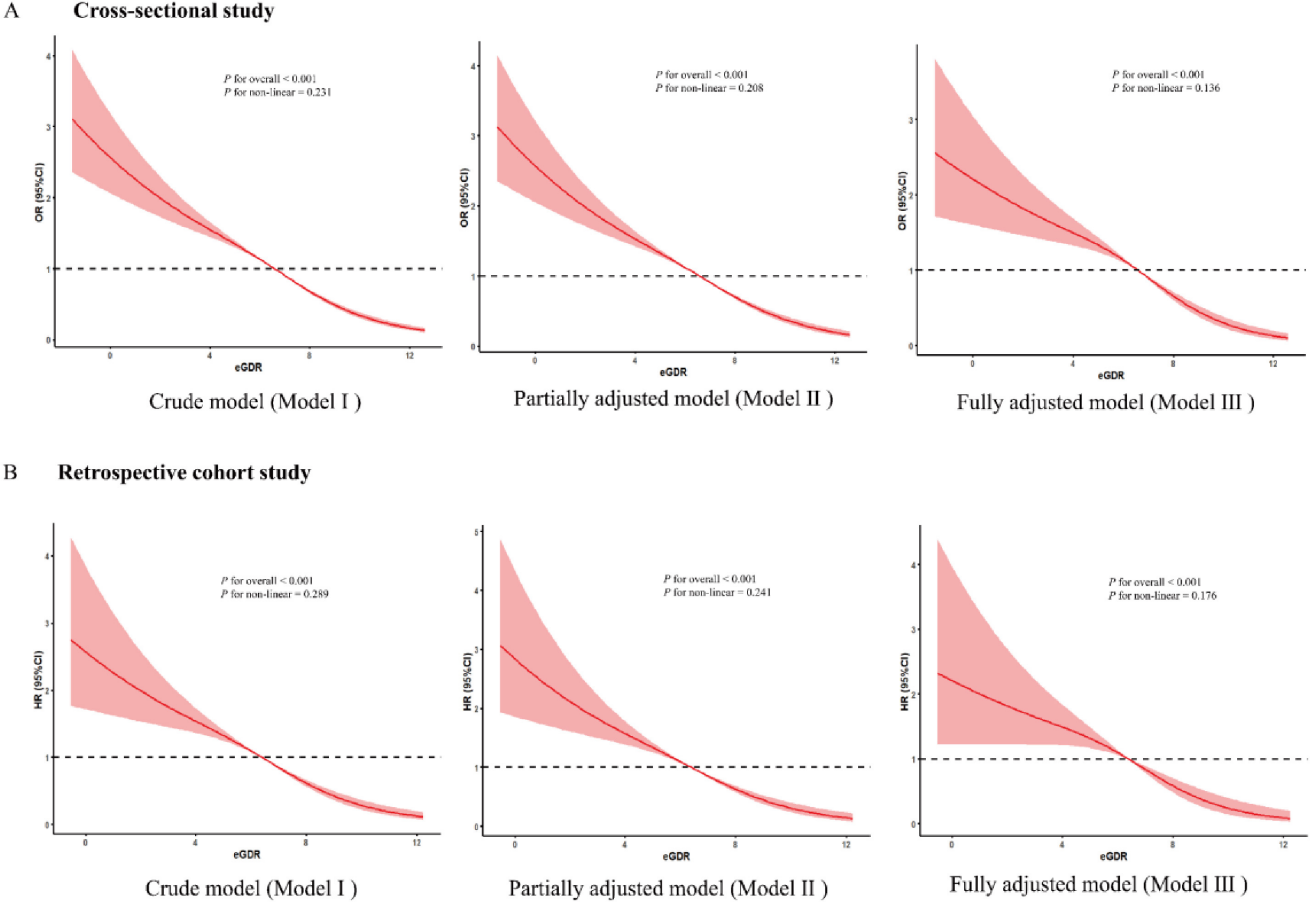
The dose-response association between eGDR and the risk of ASCVD. (A) The dose-response relationship between eGDR and ASCVD of the three models in a cross-sectional study. (B) The dose-response relationship between eGDR and new-onset ASCVD risk of the three models in a retrospective study.

In the cross-sectional study, after adjusting for some covariates, each standardized unit increase in eGDR was associated with a significantly lower odds of ASCVD, with an odds ratio (OR) of 0.839 (OR = 0.839, 95% CI: 0.806–0.894, *p*< 0.001). In addition, the highest quartile of the eGDR group was associated with a markedly lower odds of ASCVD (OR = 0.405, 95% CI: 0.276–0.576, *p* < 0.001) compared with the lowest-quartile referent group after adjusting for some covariates (Table 2). To further explore the association between eGDR and the risk of new-onset ASCVD, we conducted a retrospective cohort study based on a large population survey. Similarly, in our retrospective cohort study, each standardized unit increase in eGDR was associated with a significantly 12.4% lower hazard ratio (HR) of new-onset ASCVD (HR = 0.876, 95% CI: 0.788–0.974, *p* = 0.001) after adjusting for some covariates. Furthermore, compared with the lowest quartile of the eGDR referent group, the HR of new-onset ASCVD in the highest quartile group markedly lowered by 58.3% (HR = 0.417, 95% CI: 0.325–0.554, *p* < 0.001) after adjusting for some covariates (Table 2). The RCS curves were conducted to assess the potential non-linear relationship between eGDR and the risk of ASCVD. Additionally, after adjusting for some covariates, RCS curves demonstrated that GDR exhibited a dose-response relationship with OR of ASCVD in the cross-sectional study (*P* for overall < 0.001, *P* for nonlinear = 0.136) and the HR of new-onset ASCVD in the retrospective cohort (*P* for overall < 0.001, *P* for nonlinear = 0.176) in Figure 3.

**TABLE 2:** Association between eGDR and the risk of ASCVD in cross-sectional and retrospective studies.

Cross-sectional study			Retrospective cohort study	
Models	OR (95% CI)	*P*-value	HR (95% CI)	*P*-value
**Model I**				
Continuous	0.757 (0.723, 0.809)	<0.001	0.823 (0.741, 0.926)	<0.001
Q1	Reference		Reference	
Q2	0.681 (0.592, 0.763)	<0.001	0.712 (0.615, 0.823)	<0.001
Q3	0.476 (0.402, 0.517)	<0.001	0.524 (0.408, 0.544)	<0.001
Q4	0.168 (0.136, 0.218)	<0.001	0.237 (0.151, 0.354)	<0.001
*P* for trend	<0.001		<0.001	
**Model II**				
Continuous	0.811 (0.795, 0.827)	<0.001	0.845 (0.766, 0.950)	<0.001
Q1	Reference		Reference	
Q2	0.673 (0.595, 0.760)	<0.001	0.745 (0.630, 0.848)	<0.001
Q3	0.488 (0.429, 0.556)	<0.001	0.551 (0.425, 0.603)	<0.001
Q4	0.188 (0.159, 0.222)	<0.001	0.289 (0.202, 0.399)	<0.001
*P* for trend	<0.001		<0.001	
**Model III**				
Continuous	0.839 (0.806, 0.894)	<0.001	0.876 (0.788, 0.974)	0.001
Q1	Reference		Reference	
Q2	0.928 (0.792, 1.089)	0.361	0.804 (0.698, 0.957)	<0.001
Q3	0.760 (0.595, 0.970)	0.027	0.638 (0.521, 0.749)	<0.001
Q4	0.405 (0.276, 0.576)	<0.001	0.417 (0.325, 0.554)	<0.001
*P* for trend	<0.001		<0.001	

Q, quartile; OR, odds ratio; HR, hazard ratio. The number and concentration range of Q1–Q4 were Q1 (2,492, 1.23–4.82), Q2 (2,484, 4.83–6.59), Q3 (2,478 and 6.60–9.34), and Q4 (2,484, 9.35–12.91), respectively. Q1 was regarded as the reference group. Model I: crude model. Model II: adjusted for age, gender, ethnicity, education levels, and PIR based on Model I. Model III: adjusted for all covariates, except for WC, hypertension, and HbA1c. *P* < 0.05 was regarded as statistically significant.

### 3.3 Subgroup analyses

In this study, to validate the consistency of the negative association between eGDR and the risk of ASCVD, we conducted subgroup analyses stratified by key sociodemographic and lifestyle characteristics, including age, gender, ethnicity, etc., in our cross-sectional and retrospective cohort studies. As detailed in Table 3 and Supplementary Table 3, in multivariable-adjusted logistic regression and Cox proportional hazard regression analyses, negative associations between eGDR and the risk of ASCVD persisted across most prespecified subgroups after adjusting for some covariates, indicating a stable effect of eGDR for ASCVD risk regardless of population characteristics. Notably, subgroup analyses also revealed no significant effect modification by these population characteristics on the association between eGDR and the risk of ASCVD (all *P* for interaction > 0.05).

**TABLE 3:** Associations of eGDR with ASCVD in various subgroups in our cross-sectional study.

Subgroups	OR (95% CI)	*P*-value	*P-*adjusted	*P*-for interaction
Age				0.438
<60 years	0.842 (0.792, 0.895)	<0.001	<0.001	
≥60 years	0.847 (0.779, 0.921)	<0.001	<0.001	
Gender				0.642
Female	0.849 (0.790, 0.912)	<0.001	<0.001	
Male	0.844 (0.789, 0.901)	<0.001	<0.001	
Educational levels				0.184
Less than high-school	0.873 (0.774, 0.984)	0.012	0.039	
High school	0.856 (0.777, 0.943)	0.002	0.013	
College or above	0.833 (0.781, 0.888)	<0.001	<0.001	
Poverty index ratio				0.084
PIR < 1	0.895 (0.799, 1.003)	0.056	0.118	
1 ≤ PIR < 3	0.849 (0.789, 0.913)	<0.001	<0.001	
PIR ≥ 3	0.815 (0.751, 0.883)	<0.001	<0.001	
BMI				0.235
Underweight	0.143 (0.018, 1.118)	0.064	0.135	
Normal weight	0.564 (0.465, 0.683)	<0.001	<0.001	
Overweight	0.736 (0.653, 0.829)	<0.001	<0.001	
Obesity	0.910 (0.860, 0.962)	0.001	0.008	
Drinking status				0.378
Never drinker	0.871 (0.739, 1.027)	0.101	0.187	
Former drinker	0.834 (0.753, 0.924)	0.001	0.008	
Current drinker	0.844 (0.798, 0.892)	<0.001	<0.001	
Smoking status				0.612
Never smoker	0.866 (0.811, 0.925)	<0.001	<0.001	
Former smoker	0.841 (0.772, 0.916)	<0.001	<0.001	
Current smoker	0.788 (0.706, 0.880)	<0.001	<0.001	
Physical activity				0.732
Vigorous level	0.790 (0.725, 0.861)	<0.001	<0.001	
Middle level	0.858 (0.786, 0.935)	0.001	0.008	
Low level	0.873 (0.812, 0.939)	<0.001	<0.001	
Hypertension				0.237
Yes	0.874 (0.828, 0.922)	<0.001	<0.001	
No	0.781 (0.712, 0.856)	<0.001	<0.001	
Diabetes				0.481
Yes	0.842 (0.785, 0.904)	<0.001	<0.001	
No	0.847 (0.796, 0.902)	<0.001	<0.001	

OR, odds ratio; PIR, family income-to-poverty ratio; BMI, body mass index. All covariates were adjusted in the model, except for WC, hypertension, and HbA1c. *P* < 0.05 was regarded as statistically significant. *p*-adjusted was regarded as the *P-*value performing multiple testing correction through the Bonferroni Correction Method.

### 3.4 Increased discrimination effect of eGDR on the diagnosis of ASCVD in the cross-sectional analysis

**FIGURE 4:**
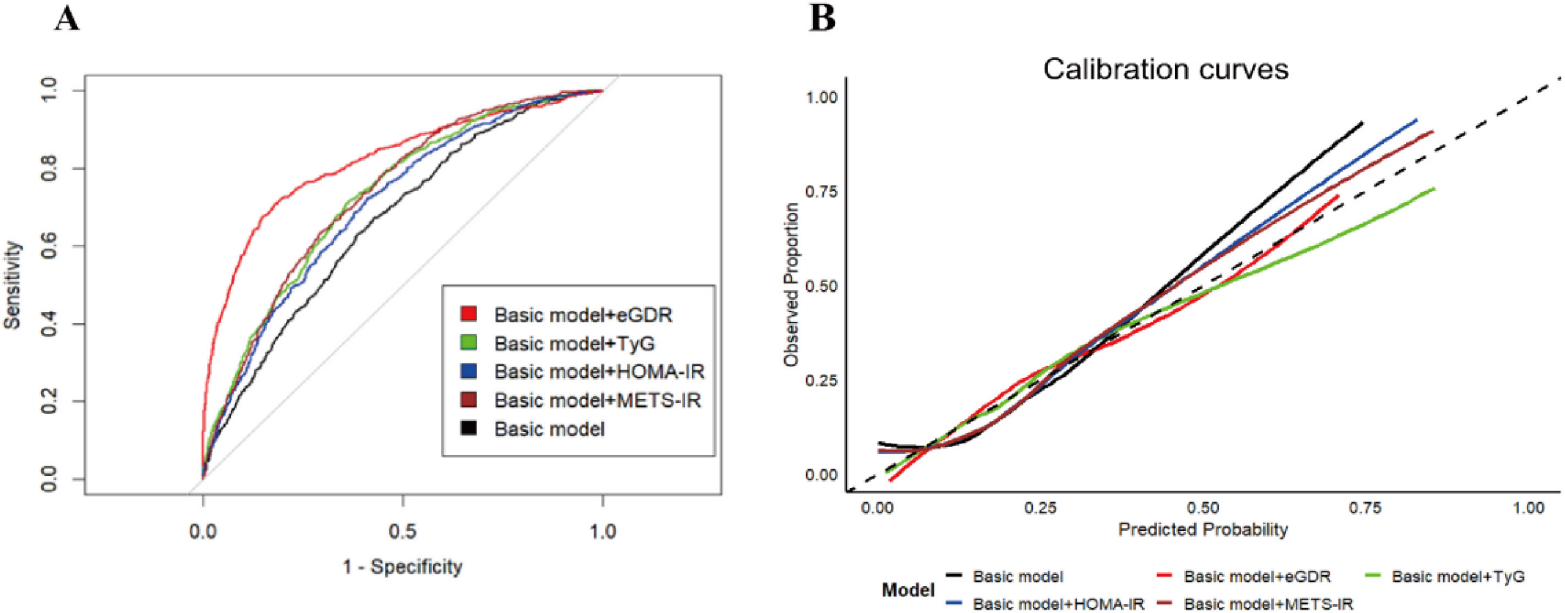
The diagnostic performance of IR-related indicators on the ASCVD diagnosis. The ROC curves (A) and calibration curves (B) for predictive performance of the ASCVD diagnosis. The basic model was adjusted for all covariates, except for WC, hypertension, and HbA1c.

Emerging evidence has established that IR-related indicators, such as TyG, HOMA-IR, and METS-IR, were associated with ASCVD (35–40). However, the diagnostic utility of eGDR, as a novel IR-related metric, for ASCVD diagnosis remained unestablished. Therefore, in this study, we employed the receiver operating characteristic (ROC) curve and some related key predictive indicators (C-statistic index, NRI, and IDI) to evaluate the diagnostic performance of eGDR on ASCVD in the cross-sectional analysis. As shown in Figure 4A and Supplementary Table 4, our results revealed that after adjusting for some covariates, all IR-related indicators have a significant diagnostic performance on the ASCVD diagnosis (all *P* < 0.05). Furthermore, eGDR presented a moderate diagnostic performance for the diagnosis of ASCVD, with the C-statistic index (0.706; 95% CI, 0.685–0.729), and had positive NRI and IDI in comparison with TyG, HOMA-IR, and METS-IR, suggesting the increased discrimination performance of eGDR on ASCVD (all *P* < 0.05). In addition, the result of the calibration curves also showed that eGDR had greater diagnostic performance compared with TyG, HOMA-IR, and METS-IR (Figure 4B).

### 3.5 Increased predictive performance of eGDR on the risk of new-onset ASCVD in the retrospective cohort analysis

**FIGURE 5:**
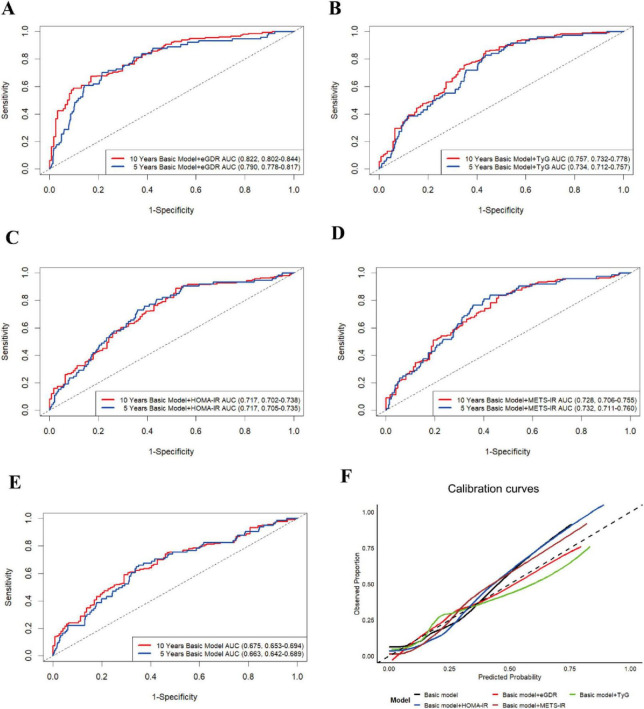
The time-dependent ROC curve (times = 5 and 10 years) for predictive effect of eGDR (A), TyG (B), HOMA-IR (C), METS-IR (D), and basic model (E) on the new-onset ASCVD risk. (F) The calibration curves (10 years) for the predictive performance of the new-onset ASCVD. The basic model was adjusted for all covariates, except for WC, hypertension, and HbA1c.

Currently, the predictive utility of eGDR, as a novel IR-related metric, for new-onset ASCVD risk remained unclear. Similarly, in our retrospective cohort analysis, we conducted the time-dependent receiver operating characteristic curve with times set at 5 and 10 years (Time-dependent ROC) and used some related key predictive indicators (C-index, NRI, and IDI) to evaluate the predictive performance of eGDR on new-onset ASCVD risk. Time-dependent ROC results demonstrated that eGDR showed a consistently moderate predictive effect on the 5-year risk (AUC = 0.790, 95% CI: 0.778–0.817) and 10-year risk (AUC = 0.822, 95% CI: 0.802–0.844) of new-onset ASCVD (Figures 5A–E). In addition, as presented in Supplementary Table 5, eGDR had the highest C-index of 0.792 (95% CI: 0.766, 0.828) and positive NRI and IDI compared to TyG, HOMA-IR, and METS-IR (all *P* < 0.05), indicating eGDR improved risk reclassification of new-onset ASCVD. In addition, the result of the calibration curves also revealed that eGDR had greater predictive performance compared with TyG, HOMA-IR, and METS-IR (Figure 5F).

### 3.6 The mediation effects of five obesity-related markers

**FIGURE 6:**
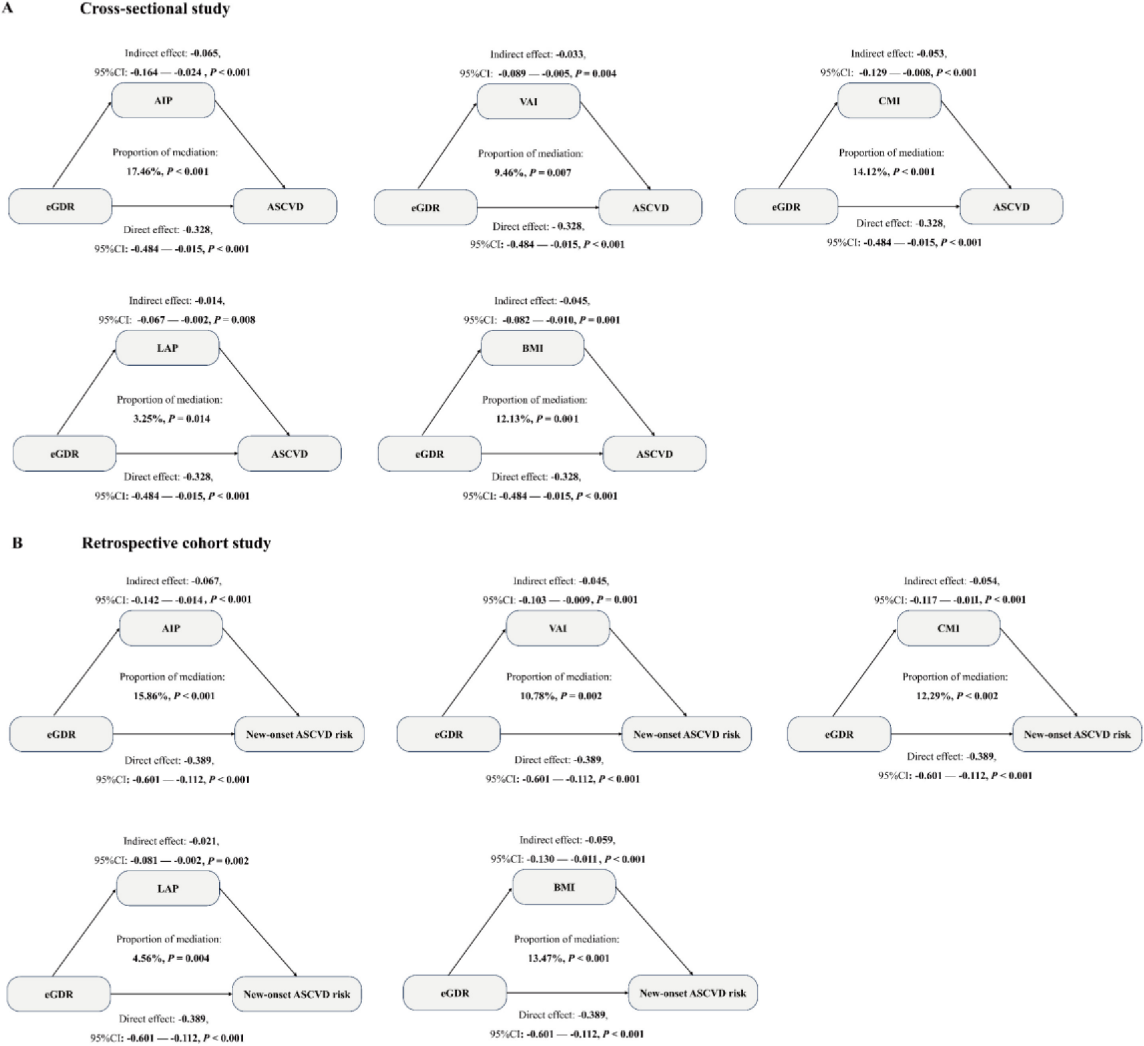
Mediation analysis was used to show the mediation effects of five related obesity indices (AIP, VAI, CMI, LAP, and BMI) in a cross-sectional study (A) and a retrospective cohort study (B). The model was adjusted for all covariates, except for WC, hypertension, and HbA1c.

The mediation analysis was conducted to explore the mediation role of five obesity-related indicators (AIP, VAI, CMI, LAP, and BMI) on the negative associations between eGDR and the risk of ASCVD in our cross-sectional and retrospective cohort study through bootstrapped samples. First, we explore the associations between eGDR and AIP, VAI, CMI, LAP, and BMI. As detailed in Supplementary Table 6, a significant negative association of eGDR with AIP, VAI, CMI, LAP, and BMI was observed (all *P* < 0.05). Then, the association of AIP, VAI, CMI, LAP, and BMI with ASCVD risk was assessed. Our results showed that five related obesity markers were positively associated with the risk of ASCVD in Supplementary Table 7 (all *P* < 0.05). Finally, the result of the mediation analysis illustrated that mediation effects of AIP, VAI, CMI, LAP, and BMI on the association between eGDR and the risk of ASCVD, with the mediated proportion of 17.40 and 15.86% for AIP, 9.46 and 10.78% for VAI, 14.12 and 12.29% for CMI, 3.25 and 4.56% for LAP, and 12.13 and 13.47% for BMI, in the cross-sectional study and the retrospective cohort study, respectively (Figure 6). These results suggested that the association was partially mediated by obesity. In addition, we performed the exposure-mediator interaction to explore their potential interactive effect, and our results showed that no exposure-mediator interaction was observed in Supplementary Table 8 (all *P* > 0.05).

### 3.7 Sensitivity analyses

To evaluate the consistency of the observed negative relationship of eGDR with the risk of ASCVD, various sensitivity analyses were performed. Since excessive missing covariate data might impact the association between eGDR and ASCVD risk, we handled these missing covariate data by multiple imputation methods in our cross-sectional and retrospective analysis, and the results demonstrated that the negative association between eGDR and ASCVD risk remained robust after imputation of these missing covariate data (Supplementary Table 9). To avoid the impact of nutritional factors on this association, we have additionally included some nutritional factors, such as energy intake, macronutrient compositions in the diet (intake of saturated fats, refined carbohydrates, and fiber), into our models. Our results showed that the negative association was robust (Supplementary Table 10). To reduce the impact of comorbidity on this relationship, we deleted participants with diabetes, chronic kidney disease, depression, and cancers in the cross-sectional and retrospective cohort analysis. These results also demonstrated that eGDR was negatively associated with the risk of ASCVD (Supplementary Table 12).

Since the applied eGDR equation was first confirmed on type 1 diabetes cohorts and is yet to be completely confirmed in the overall Chinese population. To justify the establishment of the eGDR in the Chinese general population, we added other factors (e.g., BMI) to the formula of eGDR to explore this association. Our findings revealed that this association remains negative (Supplementary Table 11). To minimize the effect of death with non-ASCVD on this association in the retrospective cohort study, we used the Fine-Gray competing-risk model, in which deaths with non-ASCVD were treated as competing-risk events, and our results showed that this association was consistent with the main results after treating death with non-ASCVD as competing-risk events (Supplementary Table 13). To lower the effect of new-onset ASCVD itself on metabolic markers such as HbA1c, blood pressure, and adiposity, leading to lower eGDR values after disease onset. We deleted new-onset ASCVD during our first follow-up to reperform this association between eGDR and new-onset ASCVD risk, and our results showed that this association was consistent with the main results (Supplementary Table 14).

## 4 Discussion

Epidemiological data have consistently demonstrated a close correlation between abnormal glucose and insulin levels and the risk of cardiovascular disease, such as heart attack, angina, heart failure, and stroke (41). Furthermore, accumulating clinical evidence indicates that elevated levels of IR biomarkers, including TyG, HOMA-IR, and METS-IR, are remarkably linked with increased cardiovascular disease risk. Individuals with cardiovascular disease exhibit markedly higher concentrations of IR-related markers than those without the disease. Mechanically, IR-related markers could increase endothelial dysfunction by activating NF-κB or PI3K/Akt signaling pathways, increasing reactive oxygen species (ROS) production, and secreting proinflammatory cytokines (TNF-α, IL-6, IL-1β), to further improve cardiovascular disease risk. In addition, some IR-related markers also impact the thrombotic status by upregulation of PAI-1 and aggregation of platelets to increase the risk of cardiovascular disease. eGDR is a novel IR indicator that integrates multidimensional metabolic parameters, including WC, hypertension status, and HbA1c, which is more accurate in response to the glucose metabolic levels and cost-effective in clinical practice. Currently, the association of eGDR with the risk of ASCVD remains unclear, and the potential mechanisms are not illustrated.

In this study, we used population health examination data from two health examination centers of Qingdao University Affiliated Shanghai Deji Hospital and Ningbo Medical Center Lihuili Hospital to design cross-sectional and retrospective cohort studies to investigate the relationship of eGDR with the risk of ASCVD. Our results showed that eGDR is negatively and linearly associated with ASCVD risk in the cross-sectional and retrospective cohort studies. And ASCVD risk is lower with eGDR increased after adjusting for some covariates, for example, compared to the lowest quartile of eGDR, the ASCVD risk of the highest quartile of eGDR decreased by 60%, which suggests the potential impact of public health strategies aimed at improving insulin sensitivity across the population. Conversely, the more modest hazard ratio for a single-unit decrease in eGDR reflects the incremental change in risk an individual might expect from a small change in insulin sensitivity. While this per-unit effect is modest, it suggests that achievable improvements in insulin resistance through lifestyle modification could confer meaningful risk reduction at both the individual and population level.

Additionally, this negative relationship maintains consistency in various subgroups, suggesting the association is stable and generalizable. In addition, our results were consistent with findings from Yan et al.’s nationwide prospective cohort study of China, in which eGDR was negatively associated with cardiovascular risk in general adults (42), and these findings were contrasted with Niswender et al., who found that this significant negative association could only be observed in women, and the negative association was not significant in males (43). The observed discrepancy of the association in the study of Niswender et al. likely originates from their methodological limitations, including restricted sample size and inherent design constraints of epidemiological approaches, notably the exclusion of male participants, which may introduce selection bias.

eGDR, as a novel IR-related indicator, is widely used to calculate the glucose metabolic levels in clinical studies due to its being less costly and having a more comprehensive formula for glucose utilization. When eGDR levels increased, the glucose utilization capability improved, which suggests a low risk of developing insulin resistance. Currently, the prediction performance of eGDR on ASCVD diagnosis and the risk of new-onset ASCVD remains unclear. Therefore, in our cross-sectional study, we conducted the ROC curve, calculating the C-statistic index, NRI, and IDI to assess the predictive performance of eGDR for ASCVD diagnosis. Our results showed that compared to TyG, HOMA-IR, and METS-IR, eGDR had a moderate predictive value on ASCVD diagnosis with the positive Δ C-statistic index, NRI, and IDI (all *P* < 0.05). Additionally, in our retrospective cohort study, we conducted the time-dependent ROC curve (times = 5 and 10 years) and calculated the C-index, NRI, and IDI to evaluate the prediction performance of eGDR on the risk of new-onset ASCVD. Our findings also demonstrated that compared to TyG, HOMA-IR, and METS-IR, eGDR had the greatest predictive effects on the risk of new-onset ASCVD with positive Δ C-index, NRI, and IDI (all *P* < 0.05). These results suggested eGDR is a more valuable IR biomarker in predicting ASCVD diagnosis and new-onset ASCVD risk, offering novel insights into ASCVD prevention strategies through improving glucose utilization capability and insulin sensitivity.

Currently, the potential mechanisms of the association of eGDR with the risk of ASCVD are not fully clear. Accumulated epidemiological studies have consistently shown a close link between eGDR and obesity (44, 45). For example, in a case-control study from China (*N* = 418 adults), Yang et.al demonstrated that the prevalence of obesity in adults decreased as eGDR levels increased (46). Furthermore, some studies have demonstrated that obesity status could impact the prevalence and incidence risk of ASCVD. Asztalos et al. revealed that after controlling for some confounding variables, increased BMI levels could independently correlate with an increase in ASCVD risk in a large cohort study from the USA (*N* = 226,000 middle-aged and elderly populations) (47). Consequently, we hypothesize that obesity may play a vital mediating role in the association between eGDR and the risk of ASCVD. However, there are no studies to explore the mediating effect of related obesity markers in the relationship of eGDR with ASCVD risk. Therefore, in our study, we explored the association between five obesity-related indices (AIP, VAI, CMI, LAP, and BMI) and ASCVD and eGDR and performed mediation analysis to evaluate the role of AIP, VAI, CMI, LAP, and BMI in the association between eGDR and ASCVD risk. Our findings demonstrated that AIP, VAI, CMI, LAP, and BMI were significantly negatively associated with eGDR and positively associated with ASCVD risk. Furthermore, AIP, VAI, CMI, LAP, and BMI had significant mediation effects (all *P* < 0.05), which suggested eGDR was negatively associated with ASCVD risk by partially mediating obesity status. Some biological evidence has supported our results. Low levels of eGDR indicate insulin resistance, which readily leads to visceral fat accumulation (48). Excessive visceral fat accumulation further triggers adipose tissue dysfunction and lipotoxicity, initiating a complex cascade of events including systemic chronic inflammation, dysregulated adipokine secretion, residual cholesterol accumulation, mitochondrial dysfunction, and endoplasmic reticulum stress (49–52). Ultimately, these processes contribute to the onset and progression of ASCVD.

Our study has several strengths. First, this study shows the moderate predictive efficacies of eGDR for ASCVD diagnosis and the risk of new-onset ASCVD compared with IR-related markers (TyG, HOMA-IR, METS-IR). Second, various potential confounding factors are adjusted to reduce the effect of these covariates on this relationship. Third, various sensitivity analyses are performed to examine the robustness of the results. Finally, this study presents that the obesity-related indices have partial mediation effects on the association between eGDR and ASCVD risk.

There are some limitations to our study. First, in our cross-sectional and retrospective cohort study, some potential confounding factors, such as unmeasured genetic predispositions (e.g., polygenic risk scores for ASCVD), were not adjusted, which may introduce bias in the observed associations. Second, in our retrospective cohort study, some individuals may be lost due to missing, death, etc., during the period of follow-up, which may affect the representativeness of the results. Third, the participants were recruited from health examination centers, which may lead to a “health volunteer bias.” This sample likely over-represents health-conscious individuals with better overall health profiles and access to healthcare, thereby limiting the generalizability of our absolute risk estimates to the broader Chinese adult population. This selection bias could potentially attenuate the observed associations by restricting the range of both risk factors and disease outcomes, or conversely, exaggerate them if the healthiest subset drives the association. Nevertheless, the internal relationships identified within this cohort are still informative. Future research should aim to replicate these findings in more representative, population-based samples to confirm their external validity. Fourth, our study shows the greater predictive effect of eGDR compared with other related IR indices; however, we do not evaluate the predictive performance of eGDR against established ASCVD risk scores such as the China-PAR model. Fifth, the eGDR exposure metric is itself a composite of waist circumference and hypertension statusture research should aim ons by retors for ASCVD. This may create a tautological loop where the predictor and outcome are not sufficiently independent. Sixthly, we revealed the mechanism that obesity partially mediated this association between eGDR and ASCVD. However, there are other factors to affect the association of eGDR with ASCVD. Finally, our study could not reveal the causal relationship of eGDR with the risk of ASCVD. Therefore, in the future, well-designed prospective longitudinal cohort studies and mechanistic animal experiments will be prioritized to establish causal relationships and elucidate the underlying pathophysiological mechanisms.

## 5 Conclusion

Our cross-sectional and retrospective cohort studies reveal a significant negative dose-response linear relationship between eGDR and the risk of ASCVD. Additionally, compared to TyG, HOMA-IR, and METS-IR, eGDR demonstrates moderate diagnostic capacities for ASCVD diagnosis and predictive capacities for the risk of new-onset ASCVD. In addition, the AIP, VAI, CMI, LAP, and BMI partially mediated the association between eGDR and the risk of ASCVD. In summary, this study elucidates the negative association of eGDR with ASCVD risk, and AIP, VAI, CMI, LAP, and BMI may partially mediate the negative association, advancing the importance of enhancing glucose utilization capability in lowering the risk of cardiovascular disease.

## Data Availability

The original contributions presented in this study are included in this article/Supplementary material, further inquiries can be directed to the corresponding author.
