# Supplementary material for: Association between estimated glucose disposal rate and the risk of atherosclerotic cardiovascular disease: insight from cross-sectional and retrospective cohort studies
Source: Front Nutr. 2025 Dec 10;12:1664591. doi: 10.3389/fnut.2025.1664591 (PMC12728581; doi:10.3389/fnut.2025.1664591)
Supplement: Supplementary file 1 [file Table_1.docx]

**Table S1** Baseline demographic characteristics of the participants in our retrospective cohort study

|  | Total (n = 4614), n (%) or mean (se) | New-onset ASCVD (n = 868), n (%) or mean (se) | None- ASCVD (n= 3746), n (%) or mean (se) | *P*-value |
| --- | --- | --- | --- | --- |
| Age | 46.23 (9.06) | 50.13 (11.23) | 43.15（8.19） | < 0.001 |
| Gender  Female  Male | 2209 (47.88)  2405 (52.12) | 416 (47.93)  452 (52.07) | 1793 (47.86)  1953 (52.14) | 0.011 |
| Educational levels  Less than High school  High school  College or above | 1423 (30.84)  1562 (33.85)  1629 (35.31) | 357 (41.13)  215 (24.77)  296 (34.10) | 1066 (28.46)  1347 (35.96)  1333 (35.58) | < 0.001 |
| PIR  PIR<1  1≤PIR<3  PIR≥3 | 1654 (35.85)  1269 (27.50)  1691 (36.65) | 328 (37.78)  203 (23.38)  337 (38.84) | 1326 (35.40)  1066 (28.46)  1354 (36.14) | 0.001 |
| BMI  Underweight  Normal weight  Overweight  Obesity | 168 (3.64)  2046 (44.34)  1332 (28.87)  1068 (23.15) | 83 (9.56)  391 (45.05)  226 (26.04)  168 (19.35) | 85 (2.26)  1655 (44.18)  1106 (29.52)  900 (24.04) | < 0.001 |
| Drinking status  Never drinker  Former drinker  Current drinker | 745 (16.15)  1698 (36.80)  2171 (47.05) | 146 (16.82)  205 (23.62)  517 (59.56) | 599 (15.99)  1493 (39.86)  1654 (44.15) | 0.126 |
| Smoking status  Never smoker  Former smoker  Current smoker | 878 (19.03)  1569 (34.01)  2167 (46.96) | 249 (28.69)  278 (32.03)  341 (39.28) | 629 (16.79)  1291 (34.46)  1826 (48.75) | < 0.001 |
| Physical activity  Vigorous level  Middle level  Low level | 456 (9.88)  1567 (33.96)  2591 (56.16) | 78 (8.98)  257 (29.61)  533 (61.41) | 378 (10.08)  1310 (34.97)  2058 (54.95) | < 0.001 |
| Hypertension  Yes  No | 1783 (38.64)  2831 (61.36) | 298 (34.33)  570 (65.67) | 1485 (39.64)  2261 (60.36) | < 0.001 |
| Diabetes  Yes  No | 1112 (24.10)  3502 (75.90) | 285 (32.83)  583 (67.17) | 827 (22.08)  2919 (77.92) | < 0.001 |
| eGDR (mg/kg/min) | 6.97 (2.25) | 6.39 (2.13) | 7.75 (2.46) | < 0.001 |
| WC (cm) | 99.63 (8.16) | 106.32 (8.85) | 94.58 (7.35) | < 0.001 |
| TC (mg/dL) | 227.14 (5.37) | 232.28 (5.89) | 222.19 (5.01) | < 0.001 |
| TG (mg/dL) | 137.16 (6.01) | 141.34 (6.76) | 133.97 (5.53) | < 0.001 |
| HDL (mg/dL) | 40.23 (4.79) | 37.08 (4.89) | 42.57 (4.35) | < 0.001 |
| HbA1c (%) | 5.98 (5.24) | 6.69 (5.67) | 5.21 (4.88) | < 0.001 |
| Fast glucose (mg/dL) | 96.14 (7.35) | 100.45 (8.32) | 92.42 (6.45) | < 0.001 |
| CRP (mg/L) | 2.54 (1.45) | 2.84 (1.37) | 2.37 (1.58) | < 0.001 |
| UA (mg/dL) | 5.65 (2.59) | 6.01 (2.45) | 5.34 (2.78) | < 0.001 |
| ALB (g/L) | 40.00 (4.05) | 43 (5.15) | 38 (3.34) | < 0.001 |
| ASL (mg/dL) | 235.56 (5.24) | 242.06 (5.89) | 231.38 (5.13) | < 0.001 |
| ATL (U/L) | 25.26 (5.03) | 28.05 (6.09) | 22.67 (4.69) | < 0.001 |
| GGT (U/L) | 35.04 (3.34) | 39.56 (3.59) | 32.45 (3.12) | < 0.001 |
| LDH (U/L) | 145.15 (7.81) | 148.78 (7.14) | 141.39 (8.29) | < 0.001 |

**Abbreviations:** PIR: family income-to-poverty ratio, BMI: body mass index, WC: waist circumference, TC: total cholesterol, TG: triglyceride, LDL: low-density lipoprotein cholesterol, HDL: high-density lipoprotein cholesterol, HbA1c: glycated hemoglobin A1c, CRP: C-reactive protein, IL-6: Interleukin-6, UA: uric acid, ALB: albumin, ALT: alanine aminotransferase, AST: aspartate aminotransferase, GGT: gamma-glutamyl transferase, LDH: lactate dehydrogenase, ASCVD: atherosclerotic cardiovascular disease. **Notes:** Categorical variables were presented as (%), with χ² tests comparing statistical differences between groups. Continuous variables were reported as mean ± standard error (SE), with between-group comparisons performed using Student's t-test. *P* < 0.05 was regarded as statistically significant.

**Table S2.** Baseline demographic characteristics of the included and excluded participants in the cohort study.

|  | Included population (n = 4614), n (%) or mean (se) | Expleded population (n  =5322), n (%) or mean (se) | *P*-value |
| --- | --- | --- | --- |
| Age | 46.01 (9.26) | 44.65（9.03） | 0.357 |
| Gender  Female  Male | 2296 (49.76)  2318 (50.24) | 2653 (49.85)  2669 (50.15) | 0.881 |
| Educational levels  Less than High school  High school  College or above | 1657 (35.91)  1715 (37.17)  1242 (26.92) | 1746 (32.81)  1597 (30.00)  1979 (37.19) | 0.361 |
| PIR  PIR<1  1≤PIR<3  PIR≥3 | 728 (15.77)  1856 (40.23)  2030 (44.00) | 926 (17.40)  1913 (35.95)  2483 (46.65) | 0.201 |
| BMI  Underweight  Normal weight  Overweight  Obesity | 123 (2.67)  2002 (43.39)  1586 (34.37)  903 (19.57) | 245 (4.60)  2344 (44.04)  1735 (32.60)  998 (18.76) | 0.378 |
| Drinking status  Never drinker  Former drinker  Current drinker | 546 (11.83)  1705 (36.95)  2363 (51.22) | 699 (13.13)  2273 (42.71)  2350 (44.16) | 0.206 |
| Smoking status  Never smoker  Former smoker  Current smoker | 599 (12.98)  2274 (49.28)  1741 (37.73) | 880 (16.54)  2014 (37.84)  2428 (45.62) | < 0.001 |
| Physical activity  Vigorous level  Middle level  Low level | 668 (14.48)  2444 (52.97)  1502 (32.55) | 788 (14.81)  2575 (48.38)  1959 (36.81) | 0.238 |
| Hypertension  Yes  No | 1608 (34.85)  3006 (65.15) | 1105 (20.76)  4217 (79.24) | < 0.001 |
| Diabetes  Yes  No | 1485 (32.18)  2129 (67.82) | 927 (17.42)  4395 (82.58) | < 0.001 |
| eGDR (mg/kg/min) | 6.96 (2.30) | 6.43 (2.36) | 0.278 |
| WC (cm) | 99.01 (7.73) | 97.71 (7.45) | 0.313 |
| TC (mg/dL) | 226.00 (5.14) | 224.78 (4.89) | 0.269 |
| TG (mg/dL) | 138.34 (6.76) | 137.86 (5.53) | 0.378 |
| HDL (mg/dL) | 37.08 (4.89) | 37.58. (4.65) | 0.428 |
| HbA1c (%) | 6.69 (5.67) | 6.01 (4.88) | 0.489 |
| Fast glucose (mg/dL) | 99.45 (8.32) | 98.52 (6.45) | 0.514 |
| CRP (mg/L) | 2.91 (1.37) | 2.44 (1.58) | 0.598 |
| UA (mg/dL) | 6.23 (2.63) | 5.56 (2.88) | 0.612 |
| ALB (g/L) | 44 (5.58) | 42 (5.34) | 0.628 |
| ASL (mg/dL) | 242.46 (5.39) | 241.05 (5.28) | 0.497 |
| ATL (U/L) | 27.15 (6.16) | 26.79 (6.28) | 0.518 |
| GGT (U/L) | 38.76 (3.79) | 37.56 (3.47) | 0.619 |
| LDH (U/L) | 146.14 (8.06) | 147.29 (7.18) | 0.419 |

**Abbreviations:** PIR: family income-to-poverty ratio, BMI: body mass index, WC: waist circumference, TC: total cholesterol, TG: triglyceride, LDL: low-density lipoprotein cholesterol, HDL: high-density lipoprotein cholesterol, HbA1c: glycated hemoglobin A1c, CRP: C-reactive protein, IL-6: Interleukin-6, UA: uric acid, ALB: albumin, ALT: alanine aminotransferase, AST: aspartate aminotransferase, GGT: gamma-glutamyl transferase, LDH: lactate dehydrogenase, ASCVD: atherosclerotic cardiovascular disease. **Notes:** Categorical variables were presented as (%), with χ² tests comparing statistical differences between groups. Continuous variables were reported as mean ± standard error (SE), with between-group comparisons performed using Student's t-test. *P* < 0.05 was regarded as statistically significant.

**Table S3.** Associations of eGDR with new-onset ASCVD risk in various subgroups in our retrospective cohort study

|  | HR (95%CI) | *P*-value | *P-*adjusted | *P* for interaction |
| --- | --- | --- | --- | --- |
| Age  < 60 years  ≥ 60 years | 0.883 (0.801, 0.974)  0.821 (0.734, 0.942) | < 0.001  < 0.001 | < 0.001  < 0.001 | 0.476 |
| Gender  Female  Male | 0.873 (0.805, 0.980)  0.892 (0.812, 0.988) | < 0.001  < 0.001 | < 0.001  < 0.001 | 0.643 |
| Educational levels  Less than High school  High school  College or above | 0.903 (0.814, 0.997)  0.874 (0.787, 0.962)  0.862 (0.795, 0.979) | <0.001  <0.001 0.001 | <0.001  <0.001  0.008 | 0.582 |
| PIR  PIR<1  1≤PIR<3  PIR≥3 | 0.926 (0.830, 1.040)  0.892 (0.801, 0.984)  0.846 (0.763, 0.956) | 0.082  < 0.001  < 0.001 | 0.173  < 0.001  < 0.001 | 0.387 |
| BMI  Underweight  Normal weight  Overweight  Obesity | 0.943 (0.804, 1.089)  0.815 (0.698, 0.929)  0.867 (0.653, 0.987)  0.965 (0.817, 1.066) | 0.156  < 0.001  < 0.001  0.105 | 0.289  < 0.001  < 0.001  0.196 | 0.376 |
| Drinking status  Never drinker  Former drinker  Current drinker | 0.803 (0.721, 0.897)  0.835 (0.743, 0.925)  0.889 (0.797, 0.998) | <0.001  <0.001  0.004 | <0.001  <0.001  0.021 | 0.126 |
| Smoking status  Never smoker  Former smoker  Current smoker | 0.786 (0.705, 0.884)  0.828 (0.754, 0.937)  0.895 (0.802, 0.983) | < 0.001  < 0.001  < 0.001 | < 0.001  < 0.001  < 0.001 | 0.165 |
| Physical activity  Vigorous level  Middle level  Low level | 0.786 (0.705, 0.884)  0.828 (0.754, 0.937)  0.895 (0.802, 0.983) | < 0.001  < 0.001  < 0.001 | < 0.001  < 0.001  < 0.001 | 0.189 |
| Hypertension  Yes  No | 0.952 (0.867, 1.073)  0.845 (0.724, 0.986) | 0.124  < 0.001 | 0.252  < 0.001 | 0.476 |
| Diabetes  Yes  No | 0.968 (0.883, 1.103)  0.863 (0.743, 0.992) | 0.243  < 0.001 | 0.381  < 0.001 | 0.698 |

**Abbreviation:** HR: hazard ratio**.** PIR: family income-to-poverty ratio, BMI: body mass index. **Notes:** The model was adjusted for all covariates, except for WC, hypertension, and HbA1c. *P* < 0.05 was regarded as statistically significant. *P*-adjusted was regarded as the P-value performing multiple testing correction through the Bonferroni correction method.

**Table S4.** Assessment of IR-related markers discrimination for the diagnosis of ASCVD

|  | C-statistic  (95%CI) | ΔC  (95%CI) | *P*-value | NRI  (95%CI) | *P*-value | IDI  (95%CI) | *P-*value |
| --- | --- | --- | --- | --- | --- | --- | --- |
| Basic model | 0.663  (0.644,  0.683) | Reference |  | Reference |  | Reference |  |
| Basic model +eGDR | 0.818  (0.801,  0.836) | 0.155  (0.118-0.192) | < 0.001 | 0.396  (0.348, 0.444） | < 0.001 | 0.178  (0.162-  0.195) | < 0.001 |
| Basic model  +TyG | 0.727  (0.709,  0.746) | 0.064  (0.026, 0.102) | < 0.001 | 0.091 (0.059,  0.122) | < 0.001 | 0.024 (0.017, 0.031) | < 0.001 |
| Basic model  +HOMA-IR | 0.705  (0.686,  0.725) | 0.042  (0.003, 0.081) | < 0.001 | -0.002  (-0.010, 0.006) | 0.589 | -0.003  (-0.001, 0.002) | 0.123 |
| Basic model  +METS-IR | 0.732  (0.714,  0.752) | 0.069  (0.031, 0.108) | < 0.001 | 0.055 (0.028, 0.082) | < 0.001 | 0.019 (0.014,  0.025) | < 0.001 |
| Basic model +eGDR VS Basic model  +TyG | / | 0.091  (0.055-0.127) | < 0.001 | 0.297 (0.253, 0.341) | < 0.001 | 0.154 (0.142,  0.168) | < 0.001 |
| Basic model +eGDR VS Basic model  +HOMA-IR | / | 0.113  (0.076,  0.150) | < 0.001 | 0.398 (0.352, 0.446) | < 0.001 | 0.177 (0.161, 0.194) | < 0.001 |
| Basic model +eGDR VS Basic model  +METS-IR | / | 0.086  (0.049,  0.122) | < 0.001 | 0.344 (0.298, 0.391) | < 0.001 | 0.158 (0.143, 0.174) | < 0.001 |

**Abbreviations:** NRI: net reclassification improvement, IDI: integrated discrimination improvement. **Notes:** Basic model was adjusted for all covariates, except for WC, hypertension, and HbA1c. *P* < 0.05 was regarded as statistically significant.

**Table S5.** Evaluation of IR-related markers discrimination for the risk of new-onset ASCVD

|  | C-index  (95%CI) | ΔC  (95%CI) | *P*-value | NRI  (95%CI) | *P*-value | IDI  (95%CI) | *P-*value |
| --- | --- | --- | --- | --- | --- | --- | --- |
| Basic model | 0.667  (0.633, 0.694) | Reference |  | Reference |  | Reference |  |
| Basic model  +eGDR | 0.792 (0.766,  0.828) | 0.125  (0.072,  0.195) | < 0.001 | 0.055 (0.020, 0.087) | 0.001 | 0.171 (0.136,  0.205) | < 0.001 |
| Basic model  +TyG | 0.735 (0.702,  0.755) | 0.068  (0.006,  0.138) | < 0.001 | 0.034  (0.008,  0.061) | 0.006 | 0.122  (0.096,  0.147) | < 0.001 |
| Basic model  +HOMA-IR | 0.713 (0.675, 0.752) | 0.046  (-0.019,  0.119) | 0.098 | 0.029  (0.006,  0.053) | 0.008 | 0.109 (0.084,  0.121) | < 0.001 |
| Basic model  +METS-IR | 0.726 (0.695, 0.757) | 0.059  (0.001,  0.124) | 0.004 | 0.024  (0.003,  0.051) | 0.009 | 0.093  (0.070,  0.115) | < 0.001 |
| Basic model  +eGDR VS Basic model  +TyG | / | 0.057 (0.011,  0.126) | < 0.001 | 0.021  (0.009,  0.040) | 0.004 | 0.090  (0.071,  0.109) | < 0.001 |
| Basic model  +eGDR VS Basic model  +HOMA-IR | / | 0.079 (0.014,  0.153) | < 0.001 | 0.026  (0.007,  0.044) | 0.006 | 0.124  (0.101,  0.146) | < 0.001 |
| Basic model  +eGDR VS Basic model  +METS-IR | / | 0.066  (0.009,  0.133) | < 0.001 | 0.030  (0.005,  0.042) | 0.006 | 0.114 (0.098,  0.126) | < 0.001 |

**Abbreviations:** NRI: net reclassification improvement, IDI: integrated discrimination improvement. ΔC: Difference of C-index **Notes:** All covariates were adjusted, except for WC, hypertension, and HbA1c, in the basic model. *P* < 0.05 was regarded as statistically significant.

**Table S6.** The association of the five related obesity indices with eGDR

|  | Cross-sectional study | | Retrospective cohort study | |
| --- | --- | --- | --- | --- |
|  | β (95%CI) | *P*-value | β (95%CI) | *P-*value |
| AIP |  |  |  |  |
| Continuous | -0.346 (-0.575, -0.148) | < 0.001 | -0.368 (-0.654, -0.167) | < 0.001 |
| Q1 | Reference |  | Reference |  |
| Q2 | -0.127 (-0.218, 0.035) | 0.189 | -0.154 (-0.234, -0.045) | < 0.001 |
| Q3 | -0.327 (-0.513, -0.176) | < 0.001 | -0.368 (-0.576, -0.195) | < 0.001 |
| Q4 | -0.657 (-0.986, -0.246) | < 0.001 | -0.723 (-0.943, -0.356) | < 0.001 |
| *P* for trend | < 0.001 |  | < 0.001 |  |
| VAI |  |  |  |  |
| Continuous | -0.113 (-0.239, -0.014) | 0.002 | -0.201 (-0.318, -0.046) | < 0.001 |
| Q1 | Reference |  | Reference |  |
| Q2 | -0.021 (-0.110, 0.034) | 0.112 | -0.135 (-0.278, -0.023) | < 0.001 |
| Q3 | -0.092 (-0.178, -0.008) | 0.009 | -0.246 (-0.412, -0.107) | < 0.001 |
| Q4 | -0.156 (-0.315, -0.016) | 0.001 | -0.587 (-0.893, -0.237) | < 0.001 |
| *P* for trend | 0.002 |  | < 0.001 |  |
| CMI |  |  |  |  |
| Continuous | -0.268 (-0.489, -0.093) | 0.001 | -0.348 (-0.563, -0.139) | < 0.001 |
| Q1 | Reference |  | Reference |  |
| Q2 | -0.063 (-0.252, 0.023) | 0.128 | -0.219 (-0.437, -0.104) | < 0.001 |
| Q3 | -0.197 (-0.412, -0.082) | 0.004 | -0.402 (-0.616, -0.217) | < 0.001 |
| Q4 | -0.379 (-0.632, -0.125) | < 0.001 | -0.678 (-0.892, -0.326) | < 0.001 |
| *P* for trend | < 0.001 |  | < 0.001 |  |
| LAP |  |  |  |  |
| Continuous | -0.063 (-0.119, -0.005) | 0.011 | -0.198 (-0.612, -0.056) | < 0.001 |
| Q1 | Reference |  | Reference |  |
| Q2 | -0.008 (-0.095, 0.044) | 0.149 | -0.134 (-0.310, -0.045) | < 0.001 |
| Q3 | -0.052 (-0.158, 0.010) | 0.065 | -0.368 (-0.578, -0.179) | < 0.001 |
| Q4 | -0.134 (-0.243, -0.009) | 0.0001 | -0.595 (-0.824, -0.256) | < 0.001 |
| *P* for trend | 0.002 |  | < 0.001 |  |
| BMI |  |  |  |  |
| Continuous | -0.243 (-0.442, -0.062) | 0.001 | -0.342 (-0.587, -0.139) | < 0.001 |
| Q1 | Reference |  | Reference |  |
| Q2 | -0.062 (-0.201, 0.036) | 0.205 | -0.159 (-0.345, -0.076) | < 0.001 |
| Q3 | -0.164 (-0.306, -0.075) | 0.003 | -0.378 (-0.562, -0.189) | < 0.001 |
| Q4 | -0.359 (-0.521, -0.176) | < 0.001 | -0.669 (-0.985, -0.387) | <b0.001 |
| *P* for trend | 0.003 |  |  |  |

**Abbreviations:** AIP: atherogenic index of plasma, VAI: visceral adiposity index, CMI: cardiometabolic index, LAP: lipid accumulation product, BMI: body mass index. **Notes:** Q1 was regarded as the reference group. The model was adjusted for all covariates, except for WC, hypertension, and HbA1c. *P* < 0.05 was regarded as statistically significant.

**Table S7.** The association between five obesity-related markers and the risk of ASCVD

|  | Cross-sectional study | | Retrospective cohort study | |
| --- | --- | --- | --- | --- |
|  | OR (95%CI) | *P*-value | HR (95%CI) | *P-*value |
| AIP |  |  |  |  |
| Continuous | 1.674 (1.139, 2.378) | < 0.001 | 1.789 (1.246, 2.479) | < 0.001 |
| Q1 | Reference |  | Reference |  |
| Q2 | 1.102 (1.023, 1.475) | 0.035 | 1.209 (1.026, 1.536) | 0.013 |
| Q3 | 1.463 (1.210, 1.758) | < 0.001 | 1.504 (1.254, 1.893) | < 0.001 |
| Q4 | 1.875 (1.329, 2.543) | < 0.001 | 2.087 (1.506, 2.765) | < 0.001 |
| *P* for trend | < 0.001 |  | < 0.001 |  |
| VAI |  |  |  |  |
| Continuous | 1.421 (1.098, 1.896) | 0.001 | 1.575 (1.178, 1.982) | < 0.001 |
| Q1 | Reference |  | Reference |  |
| Q2 | 1.064 (1.003, 1.328) | 0.047 | 1.136 (0.893, 1.563) | 0.089 |
| Q3 | 1.287 (1.095, 1.479) | 0.002 | 1.378 (1.089, 1.894) | < 0.001 |
| Q4 | 1.659 (1.189, 2.136) | <0.001 | 1.726 (1.378, 2.459) | < 0.001 |
| *P* for trend | 0.005 |  | < 0.001 |  |
| CMI |  |  |  |  |
| Continuous | 1.586 (1.183, 2.135) | <0.001 | 1.829 (1.357, 2.578) | < 0.001 |
| Q1 | Reference |  | Reference |  |
| Q2 | 1.106 (1.032, 1.459) | 0.024 | 1.356 (1.137, 2.139) | < 0.001 |
| Q3 | 1.382 (1.203, 1.696) | < 0.001 | 1.589 (1.298, 2.367) | < 0.001 |
| Q4 | 1.801 (1.315, 2.432) | < 0.001 | 1.986 (1.565, 2.789) | < 0.001 |
| *P* for trend | < 0.001 |  | < 0.001 |  |
| LAP |  |  |  |  |
| Continuous | 1.186 (1.010, 1.476) | 0.042 | 1.763 (1.321, 2.451) | < 0.001 |
| Q1 | Reference |  | Reference |  |
| Q2 | 1.014 (-0.096, 1.128) | 0.231 | 1.246 (1.109, 2.047) | < 0.001 |
| Q3 | 1.123 (1.005, 1.398) | 0.046 | 1.498 (1.245, 2.178) | < 0.001 |
| Q4 | 1.421 (1.065, 1.843) | 0.008 | 1.897 (1.478, 2.514) | < 0.001 |
| *P* for trend | 0.014 |  | < 0.001 |  |
| BMI |  |  |  |  |
| Continuous | 1.498 (1.112, 1.964) | < 0.001 | 1.853 (1.365, 2.754) | < 0.001 |
| Q1 | Reference |  | Reference |  |
| Q2 | 1.076 (1.008, 1.356) | 0.047 | 1.289 (1.106, 1.576) | < 0.001 |
| Q3 | 1.256 (1.087, 1.524) | 0.006 | 1.654 (1.301, 2.369) | < 0.001 |
| Q4 | 1.610 (1.254, 2.265) | < 0.001 | 2.342 (1.679, 2.897) | < 0.001 |
| *P* for trend | 0.005 |  | < 0.001 |  |

**Abbreviations:** AIP: atherogenic index of plasma, VAI: visceral adiposity index, CMI: cardiometabolic index, LAP: lipid accumulation product, BMI: body mass index. OR: odds ratio, HR: hazard ratio. **Notes:** Q1 was regarded as the reference group. The model was adjusted for all covariates, except for WC, hypertension, and HbA1c. *P* < 0.05 was regarded as statistically significant.

**Table S8.** Interaction analysis of eGDR and obesity-related index on ASCVD

| The interactive effect  of VAI and eGDR | OR (95%CI) | HR (95%CI) |
| --- | --- | --- |
| Multiplicative scale | 0.831 (0.540, 1.232) | 0.814 (0.591, 1.290) |
| RERI | -0.252 (-0.631, 0.135) | -0.331 (-0.753, 0.193) |
| AP | -0.120 (-0.525, 0.183) | -0.216 (-0.601, 0.262) |
| SI | 0.786 (0.467, 1.248) | 0.823 (0.402, 1.325) |
| The interactive effect  of VAI and eGDR |  |  |
| Multiplicative scale | 0.769 (0.552, 1.128) | 0.837 (0.571, 1.209) |
| RERI | -0.301 (-0.673, 0.123) | -0.361 (-0.819, 0.190) |
| AP | -0.180 (-0.531, 0.201) | -0.162 (-0.667, 0.391) |
| SI | 0.716 (0.317, 1.125) | 0.646 (0.247, 1.078) |
| The interactive effect  of CMI and eGDR |  |  |
| Multiplicative scale | 0.783 (0.512, 1.203) | 0.727 (0.424, 1.140) |
| RERI | -0.272 (-0.660, 0.144) | -0.262 (-0.703, 0.213) |
| AP | -0.190 (-0.540, 0.289) | -0.280 (-0.721, 0.312) |
| SI | 0.682 (0.306, 1.174) | 0.727 (0.246, 1.289) |
| The interactive effect  of LAP and eGDR |  |  |
| Multiplicative scale | 0.815 (0.505, 1.208) | 0.806 (0.312, 1.328) |
| RERI | -0.251 (-0.795, 0.263) | -0.17 (-0.573, 0.226) |
| AP | -0.323 (-0.831, 0.251) | -0.234 (-0.785, 0.361) |
| SI | 0.734 (0.277, 1.295) | 0.828 (0.207, 1.425) |
| The interactive effect  of LAP and eGDR |  |  |
| Multiplicative scale | 0.769 (0.582, 1.128) | 0.836 (0.403, 1.302) |
| RERI | -0.364 (-0.813, 0.304) | -0.290 (-0.783, 0.306) |
| AP | -0.317 (-0.789, 0.328) | -0.326 (-0.879, 0.316) |
| SI | 0.806 (0.267, 1.318) | 0.791 (0.207, 1.510) |

**Abbreviations:** RERI: relative excess risk due to interaction, AP: attributable proportion due to interaction, SI: synergy index. OR: odds ratio, HR: hazard ratio. **Notes:** The model was adjusted for all covariates, except for WC, hypertension, and HbA1c. *P* < 0.05 was regarded as statistically significant.

**Table S9.** Association between eGDR and ASCVD risk after imputing missing covariates by multiple imputation methods.

|  | Cross-sectional study | | Retrospective cohort study | |
| --- | --- | --- | --- | --- |
|  | OR (95%CI) | *P*-value | HR (95%CI) | *P*-value |
| Model Ⅰ |  |  |  |  |
| Continuous | 0.816 (0.771, 0.823) | < 0.001 | 0.830 (0.775, 0.897) | < 0.001 |
| Q1 | Reference |  | Reference |  |
| Q2 | 0.682 (0.612, 0.783) | < 0.001 | 0.699 (0.624, 0.781) | < 0.001 |
| Q3 | 0.478 (0.419, 0.532) | < 0.001 | 0.492 (0.421, 0.568) | < 0.001 |
| Q4 | 0.173 (0.142, 0.204) | < 0.001 | 0.217 (0.169, 0.292) | < 0.001 |
| *P* for trend | < 0.001 |  | < 0.001 |  |
| Model Ⅱ |  |  |  |  |
| Continuous | 0.834 (0.787, 0.861) | < 0.001 | 0.879 (0.825, 0.948) | < 0.001 |
| Q1 | Reference |  | Reference |  |
| Q2 | 0.694 (0.620, 0.792) | < 0.001 | 0.758 (0.683, 0.838) | < 0.001 |
| Q3 | 0.495 (0.437, 0.583) | < 0.001 | 0.543 (0.472, 0.627) | < 0.001 |
| Q4 | 0.197 (0.172, 0.235) | < 0.001 | 0.255 (0.198, 0.323) | < 0.001 |
| *P* for trend | < 0.001 |  | < 0.001 |  |
| Model Ⅲ |  |  |  |  |
| Continuous | 0.865 (0.811, 0.907) | < 0.001 | 0.912 (0.857, 0.975) | < 0.001 |
| Q1 | Reference |  | Reference |  |
| Q2 | 0.934 0.782 1.098 | 0.387 | 0.867 (0.811, 0.974) | < 0.001 |
| Q3 | 0.765 0.612 0.986 | 0.031 | 0.652 (0.579, 0.761) | < 0.001 |
| Q4 | 0.411 0.283 0.583 | < 0.001 | 0.322 (0.249, 0.436) | < 0.001 |
| *P* for trend | < 0.001 |  | < 0.001 |  |

**Abbreviations:** OR: odds ratio, HR: hazard ratio. **Notes:** Q1 was regarded as the reference group. All covariates were adjusted, except for WC, hypertension, and HbA1c, in the model. *P* < 0.05 was regarded as statistically significant.

**Table S10.** Association between eGDR and ASCVD risk after additionally adjusting for some nutritional factors, such as energy intake, macronutrient compositions in the diet ( intake of saturated fats, refined carbohydrates, and fiber)

|  | Cross-sectional study | | Retrospective cohort study | |
| --- | --- | --- | --- | --- |
|  | OR (95%CI) | *P*-value | HR (95%CI) | *P*-value |
| Model Ⅰ |  |  |  |  |
| Continuous | 0.827 (0.763, 0.879) | < 0.001 | 0.847 (0.792, 0.906) | < 0.001 |
| Q1 | Reference |  | Reference |  |
| Q2 | 0.670 (0.603, 0.765) | < 0.001 | 0.691 (0.614, 0.792) | < 0.001 |
| Q3 | 0.494 (0.421, 0.579) | < 0.001 | 0.512 (0.443, 0.593) | < 0.001 |
| Q4 | 0.225 (0.142, 0.294) | < 0.001 | 0.267 (0.181, 0.342) | < 0.001 |
| *P* for trend | < 0.001 |  | < 0.001 |  |
| Model Ⅱ |  |  |  |  |
| Continuous | 0.860 (0.804, 0.912) | < 0.001 | 0.883 (0.835, 0.932) | < 0.001 |
| Q1 | Reference |  | Reference |  |
| Q2 | 0.704 (0.632, 0.789) | < 0.001 | 0.764 (0.681, 0.858) | < 0.001 |
| Q3 | 0.525 (0.447, 0.596) | < 0.001 | 0.586 (0.494, 0.670) | < 0.001 |
| Q4 | 0.247 (0.173, 0.315) | < 0.001 | 0.305 (0.218, 0.382) | < 0.001 |
| *P* for trend | < 0.001 |  | < 0.001 |  |
| Model Ⅲ |  |  |  |  |
| Continuous | 0.897 (0.836, 0.947) | < 0.001 | 0.914 (0.867, 0.983) | < 0.001 |
| Q1 | Reference |  | Reference |  |
| Q2 | 0.739 (0.682, 0.856) | < 0.001 | 0.792 (0.715, 0.904) | < 0.001 |
| Q3 | 0.587 (0.503, 0.675) | < 0.001 | 0.665 (0.599, 0.782) | < 0.001 |
| Q4 | 0.325 (0.283, 0.421) | < 0.001 | 0.351 (0.267, 0.458) | < 0.001 |
| *P* for trend | < 0.001 |  | < 0.001 |  |

**Abbreviations:** OR: odds ratio, HR: hazard ratio. **Notes:** Q1 was regarded as the reference group. All covariates were adjusted, except for WC, hypertension, and HbA1c, in the model. *P* < 0.05 was regarded as statistically significant.

**Table S11.** Association between eGDR and ASCVD risk after adding BMI to the formula of eGDR

|  | Cross-sectional study | | Retrospective cohort study | |
| --- | --- | --- | --- | --- |
|  | OR (95%CI) | *P*-value | HR (95%CI) | *P*-value |
| Model Ⅰ |  |  |  |  |
| Continuous | 0.768 (0.713, 0.844) | < 0.001 | 0.787 (0.721, 0.864) | < 0.001 |
| Q1 | Reference |  | Reference |  |
| Q2 | 0.630 (0.523, 0.745) | < 0.001 | 0.651 (0.604, 0.762) | < 0.001 |
| Q3 | 0.524 (0.461, 0.589) | < 0.001 | 0.542 (0.483, 0.613) | < 0.001 |
| Q4 | 0.315 (0.242, 0.395) | < 0.001 | 0.335 (0.263, 0.414) | < 0.001 |
| *P* for trend | < 0.001 |  | < 0.001 |  |
| Model Ⅱ |  |  |  |  |
| Continuous | 0.813 (0.746, 0.887) | < 0.001 | 0.832 (0.743, 0.918) | < 0.001 |
| Q1 | Reference |  | Reference |  |
| Q2 | 0.684 (0.612, 0.764) | < 0.001 | 0.732 (0.670, 0.828) | < 0.001 |
| Q3 | 0.553 (0.485, 0.615) | < 0.001 | 0.596 (0.512, 0.684) | < 0.001 |
| Q4 | 0.348 (0.273, 0.425) | < 0.001 | 0.362 (0.287, 0.432) | < 0.001 |
| *P* for trend | < 0.001 |  | < 0.001 |  |
| Model Ⅲ |  |  |  |  |
| Continuous | 0.847 (0.806, 0.905) | < 0.001 | 0.867 (0.804, 0.938) | < 0.001 |
| Q1 | Reference |  | Reference |  |
| Q2 | 0.719 (0.652, 0.804) | < 0.001 | 0.750 (0.685, 0.874) | < 0.001 |
| Q3 | 0.602 (0.533, 0.697) | < 0.001 | 0.655 (0.546, 0.763) | < 0.001 |
| Q4 | 0.415 (0.313, 0.493) | < 0.001 | 0.431 (0.346, 0.517) | < 0.001 |
| *P* for trend | < 0.001 |  | < 0.001 |  |

**Abbreviations:** OR: odds ratio, HR: hazard ratio. **Notes:** Q1 was regarded as the reference group. All covariates were adjusted, except for WC, hypertension, and HbA1c, in the model. *P* < 0.05 was regarded as statistically significant.

**Table S12.** Association between eGDR and ASCVD after deleting precipitants with diabetes and chronic kidney disease, depression, and cancer.

|  | Cross-sectional study | | Retrospective cohort study | |
| --- | --- | --- | --- | --- |
|  | OR (95%CI) | *P*-value | HR (95%CI) | *P*-value |
| Model Ⅰ |  |  |  |  |
| Continuous | 0.823 (0.768, 0.874) | < 0.001 | 0.879 (0.837, 0.934) | < 0.001 |
| Q1 | Reference |  | Reference |  |
| Q2 | 0.624 (0.512, 0.731) | < 0.001 | 0.701 (0.632, 0.784) | < 0.001 |
| Q3 | 0.523 (0.438, 0.625) | < 0.001 | 0.587 (0.479, 0.672) | < 0.001 |
| Q4 | 0.275 (0.189, 0.359) | < 0.001 | 0.324 (0.266, 0.396) | < 0.001 |
| *P* for trend | < 0.001 |  | < 0.001 |  |
| Model Ⅱ |  |  |  |  |
| Continuous | 0.873 (0.812, 0.938) | < 0.001 | 0.909 (0.852, 0.968) | < 0.001 |
| Q1 | Reference |  | Reference |  |
| Q2 | 0.657 (0.676, 0.858) | < 0.001 | 0.779 (0.713, 0.871) | < 0.001 |
| Q3 | 0.574 (0.476, 0.649) | < 0.001 | 0.602 (0.543, 0.683) | < 0.001 |
| Q4 | 0.286 (0.197, 0.376) | < 0.001 | 0.358 (0.295, 0.439) | < 0.001 |
| *P* for trend | < 0.001 |  | < 0.001 |  |
| Model Ⅲ |  |  |  |  |
| Continuous | 0.901 (0.827, 0.976) | 0.012 | 0.935 (0.883, 0.995) | 0.022 |
| Q1 | Reference |  | Reference |  |
| Q2 | 0.853 (0.739, 1.001) | 0.052 | 0.823 (0.769, 0.914) | < 0.001 |
| Q3 | 0.713 (0.619, 0.898) | < 0.001 | 0.658 (0.586, 0.764) | < 0.001 |
| Q4 | 0.439 (0.215, 0.675) | < 0.001 | 0.469 (0.347, 0.617) | < 0.001 |
| *P* for trend | < 0.001 |  | < 0.001 |  |

**Abbreviations:** OR: odds ratio, HR: hazard ratio. **Notes:** Q1 was regarded as the reference group. All covariates were adjusted, except for WC, hypertension, and HbA1c, in the model. *P* < 0.05 was regarded as statistically significant.

**Table S13.** Association between eGDR and ASCVD risk, treating non-ASCVD death as a competing risk event in our retrospective cohort

|  | HR (95%CI) | *P*-value |
| --- | --- | --- |
| Model Ⅰ |  |  |
| Continuous | 0.846 (0.800, 0.904) | < 0.001 |
| Q1 | Reference |  |
| Q2 | 0.673 (0.612, 0.762) | < 0.001 |
| Q3 | 0.512 (0.479, 0.605) | < 0.001 |
| Q4 | 0.354 (0.281, 0.436) | < 0.001 |
| *P* for trend | < 0.001 |  |
| Model Ⅱ |  |  |
| Continuous | 0.883 (0.826, 0.951) | < 0.001 |
| Q1 | Reference |  |
| Q2 | 0.729 (0.653, 0.831) | < 0.001 |
| Q3 | 0.632 (0.563, 0.693) | < 0.001 |
| Q4 | 0.383 (0.305, 0.482) | < 0.001 |
| *P* for trend | < 0.001 |  |
| Model Ⅲ |  |  |
| Continuous | 0.913 (0.851, 0.968) | < 0.001 |
| Q1 | Reference |  |
| Q2 | 0.831 (0.745, 0.897) | < 0.001 |
| Q3 | 0.649 (0.592, 0.734) | < 0.001 |
| Q4 | 0.428 (0.357, 0.543) | < 0.001 |
| *P* for trend | < 0.001 |  |

**Abbreviations:** HR: hazard ratio. **Notes:** Q1 was regarded as the reference group. All covariates were adjusted, except for WC, hypertension, and HbA1c, in the model. *P* < 0.05 was regarded as statistically significant.

**Table S14.** Association between eGDR and new-onset ASCVD risk in our retrospective cohort

|  | HR (95%CI) | *P*-value |
| --- | --- | --- |
| Model Ⅰ |  |  |
| Continuous | 0.866 (0.818, 0.923) | < 0.001 |
| Q1 | Reference |  |
| Q2 | 0.703 (0.634, 0.781) | < 0.001 |
| Q3 | 0.554 (0.492, 0.636) | < 0.001 |
| Q4 | 0.390 (0.325, 0.459) | < 0.001 |
| *P* for trend | < 0.001 |  |
| Model Ⅱ |  |  |
| Continuous | 0.895 (0.839, 0.960) | < 0.001 |
| Q1 | Reference |  |
| Q2 | 0.734 (0.671, 0.826) | < 0.001 |
| Q3 | 0.611 (0.546, 0.684) | < 0.001 |
| Q4 | 0.402 (0.346, 0.479) | < 0.001 |
| *P* for trend | < 0.001 |  |
| Model Ⅲ |  |  |
| Continuous | 0.926 (0.881, 0.983) | 0.001 |
| Q1 | Reference |  |
| Q2 | 0.801 (0.710, 0.895) | < 0.001 |
| Q3 | 0.652 (0.581, 0.760) | < 0.001 |
| Q4 | 0.441 (0.342, 0.550) | < 0.001 |
| *P* for trend | < 0.001 |  |

**Abbreviations:** HR: hazard ratio. **Notes:** Q1 was regarded as the reference group. All covariates were adjusted, except for WC, hypertension, and HbA1c, in the model. *P* < 0.05 was regarded as statistically significant.
